# Immunization with a Hemagglutinin-Derived Synthetic Peptide Formulated with a CpG-DNA-Liposome Complex Induced Protection against Lethal Influenza Virus Infection in Mice

**DOI:** 10.1371/journal.pone.0048750

**Published:** 2012-11-07

**Authors:** Jae Won Rhee, Dongbum Kim, Byung Kwon Park, Sanghoon Kwon, Sunhee Cho, Ilseob Lee, Man-Seong Park, Jae-Nam Seo, Yong-Sun Kim, Hong Seok Choi, Younghee Lee, Hyung-Joo Kwon

**Affiliations:** 1 Center for Medical Science Research, College of Medicine, Hallym University, Gangwon-do, Republic of Korea; 2 Department of Microbiology, College of Medicine, Hallym University, Gangwon-do, Republic of Korea; 3 Department of Pathology, College of Medicine, Hallym University, Gangwon-do, Republic of Korea; 4 Department of Biochemistry, College of Natural Sciences, Chungbuk National University, Chungbuk, Republic of Korea; University of Massachusetts Medical Center, United States of America

## Abstract

Whole-virus vaccines, including inactivated or live-attenuated influenza vaccines, have been conventionally developed and supported as a prophylaxis. These currently available virus-based influenza vaccines are widely used in the clinic, but the vaccine production takes a long time and a huge number of embryonated chicken eggs. To overcome the imperfection of egg-based influenza vaccines, epitope-based peptide vaccines have been studied as an alternative approach. Here, we formulated an efficacious peptide vaccine without carriers using phosphodiester CpG-DNA and a special liposome complex. Potential epitope peptides predicted from the hemagglutinin (HA) protein of the H5N1 A/Viet Nam/1203/2004 strain (NCBI database, AAW80717) were used to immunize mice along with phosphodiester CpG-DNA co-encapsulated in a phosphatidyl-β-oleoyl-γ-palmitoyl ethanolamine (DOPE):cholesterol hemisuccinate (CHEMS) complex (Lipoplex(O)) without carriers. We identified a B cell epitope peptide (hH5N1 HA233 epitope, 14 amino acids) that can potently induce epitope-specific antibodies. Furthermore, immunization with a complex of the B cell epitope and Lipoplex(O) completely protects mice challenged with a lethal dose of recombinant H5N1 virus. These results suggest that our improved peptide vaccine technology can be promptly applied to vaccine development against pandemic influenza. Furthermore our results suggest that potent epitopes, which cannot be easily found using proteins or a virus as an antigen, can be screened when we use a complex of peptide epitopes and Lipoplex(O).

## Introduction

Influenza A viruses have gained attention because pandemic influenza causes human diseases associated with respiratory tract complication and high mortality. Recently, several emerging influenza A subtypes such as H1N1 [1], H5N1 [2], [3], H7N7 [4], and H9N2 [5] have been shown to have originated from swine, birds or humans. The emergence of the highly pathogenic avian influenza strains of H5N1 in South-East Asia in 1997 [2] has raised the possibility of human-to-human transmission [2], [6] and new pandemic threats [7], [8].

Diverse studies have endeavored to develop prophylaxis and therapeutics against influenza viruses. Whole-virus vaccines, including inactivated or live-attenuated influenza vaccines, have been conventionally developed and supported as a prophylaxis. Inactivated influenza virus vaccines are safe and can induce significant protective neutralizing antibodies with an efficacy of 60% to 90% [9]. Several live-attenuated vaccines have been developed by means of reverse genetics technology [10], cold-adapted master strains [11], and genetically homologous attenuated strains to avoid the risk of generating pathogenic reassortants [12]–[14]. These currently available virus-based influenza vaccines are widely used in the clinic, but the vaccine production takes a long time and needs a great number of specific-pathogen-free embryonated chicken eggs. To overcome these shortcomings, several studies have investigated safe, high-speed, cell-based production of viruses [15], [16]. One alternative approach uses recombinant viral proteins, such as the hemagglutinin (HA) protein of the H5N1 virus, which are produced in cells [17]. To further potentiate the application of recombinant proteins, researchers have developed DNA vaccines [18], [19], and evaluated them in mice.

In addition, epitope-based peptide vaccines have been used as an alternative approach to improving the immunogenicity, protecting against divergent strains, shortening the production time, and reducing side effects [20]–[22]. Numerous studies have reported B cell epitopes and T cell epitopes for protecting against influenza viruses, including avian H5N1 and human influenza strains [20]–[24]. Although peptide vaccines are effective in various animal models, their efficacy is limited when applied to humans. Some attempts have been made to improve the peptide vaccine efficacy using liposomes or adjuvants such as flagella and CpG-DNA containing immunostimulatory CpG motifs [25]–[27].

Several investigators have reported that CpG-DNA has potent functional effects as a Th1-responsive adjuvant [28]. Previously, we identified the natural phosphodiester bond CpG-DNA (PO-ODN, MB-ODN 4531(O)) with immunostimulatory activity from *M. bovis* genomic DNA [29], [30]. Furthermore, we found that a complex of peptide epitope and MB-ODN 4531(O) co-encapsulated in a phosphatidyl-β-oleoyl-γ-palmitoyl ethanolamine (DOPE):cholesterol hemisuccinate (CHEMS) complex without carriers significantly enhanced peptide-specific IgG production [31]–[33]. We define MB-ODN 4531(O) co-encapsulated in a DOPE:CHEMS complex (1∶1 ratio) as Lipoplex(O). In this study, we identified a B cell epitope peptide, from the HA protein of the H5N1 A/Vietnam/1203/2004 strain, which can potently induce production of epitope-specific antibodies. In addition, we report that the immunization with a complex of B cell epitope of HA protein and Lipoplex(O) completely protected the mice from the challenge by a lethal dose of recombinant H5N1 virus (rH5N1 virus). These results suggest that epitope peptide vaccine is greatly improved by Lipoplex(O) and can be promptly applied to vaccine development in preparation against pandemic influenza.

## Results

### Selection of B cell Epitopes from H5N1 HA Protein and Induction of the Epitope-specific IgG Production by a Complex of Epitope and Lipoplex(O)

**Figure 1:**
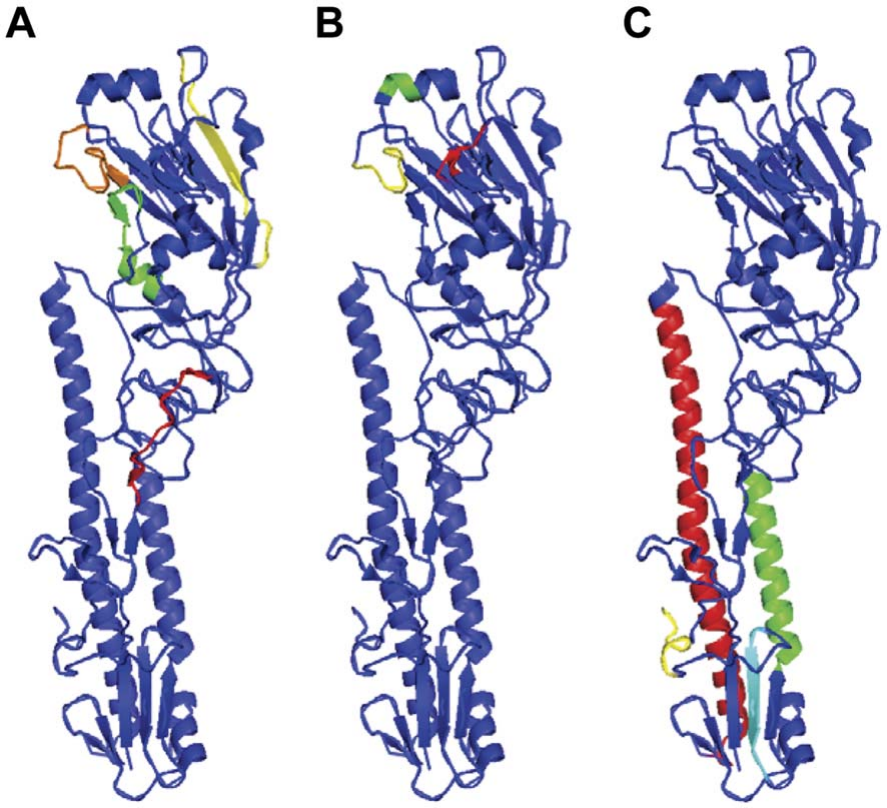
Location of B cell epitopes used in this study in the HA structure. (**A**) Schematic representation of predicted B cell epitopes used in this study. Red, hH5N1 HA58; Green, hH5N1 HA113; Yellow, hH5N1 HA175; Orange, hH5N1 HA233. (**B**) Location of receptor-binding site. Red, 130-loop; Green, 190-helix; Yellow, 220-loop. (**C**) Schematic representation of previously reported B cell epitopes. Red, LAH; Green, H5-F10 and CR6261; Yellow, H5-1C9; Cyan, H5-1B12. The image of HA structure of the A/Duck/Singapore/3/1997 strain was created with the use of PyMOL (www.pymol.org) and the HA structure was obtained from the Protein Data Bank (PDB: 1JSM).

The influenza HA is a surface glycoprotein (HA0), which is cleaved to two polypeptides, HA1 and HA2 [34]. First, four candidate peptide sequences (hH5N1 HA58, hH5N1 HA113, hH5N1 HA175, hH5N1 HA233) were selected from the HA1 polypeptide region of the H5N1 A/Vietnam/1203/2004 strain HA protein on the basis of their hydrophilicity, hydrophobicity, secondary structure, and antigenicity index as described in “Materials and Methods” (Table 1, Figure 1A). Among the candidates, hH5N1 HA233 overlaps with one of the receptor binding sites (Figure 1B). Currently, several highly conserved B cell epitopes were reported in the HA2 polypeptide region of the HA protein (Table 2, Figure 1C) [22],[35]–[37]. Therefore, we synthesized six more candidate peptide sequences (H5-F10, H5-1C9, H5-1B12, H5-LAH-1, H5-LAH-2) (Table 2). Then, we prepared a complex containing each epitope and Lipoplex(O).

**Table 1:** Candidate epitopes of A/Vietnam/1203/2004 hH5N1 HA protein.

Sequences	Location	Abbreviation
ILEKKHNGKLC	58–68	hH5N1 HA58
CYPGDFNDYEELK	113–125	hH5N1 HA113
STYTIKRSYNNTNQ	175–188	hH5N1 HA175
IATRSKVNGQSGRM	233–246	hH5N1 HA233

Peptide sequences for epitope screening were selected from HA1 polypeptide region of A/Vietnam/1203/2004 H5N1 HA protein (NCBI database, AAW80717) based on hydrophilicity, hydrophobicity, secondary structure, antigenicity index, and amphipathicity (http://tools.immuneepitpoe.org/main/index.html). Amino acid sequence of A/Vietnam/1203/2004 hH5N1 HA protein is numbered on the basis of alignment with the human H3 sequence (A/Aichi/2/68).

**Table 2:** Reported monoclonal antibodies against B cell epitopes of HA2 polypeptide derived from influenza A virus strains.

Monoclonal antibodies^a^	Epitope sequences	Sequences used in this study^b^	Abbreviations in this study^c^	References
mAb F10	^381^ADKESTQKAIDGVT NKVNSIIDK^403^	^385^STQKAIDGVTNKVNSIIDK^403^	H5-F10	(34)
mAb CR6261	^388^KAIDGVTNKVNSIIDK^403^	^385^STQKAIDGVTNKVNSIIDK^403^	H5-F10	(35)
MAb 1C9	^346^GLFGAIAGF^354^	^341^RKKRGLFGAIAGFIEGGW^360^	H5-1C9	(36)
MAb 1B12	^366^WYGYHHSN^373^	^363^VDGWYGYHHSNEQGSGYA^380^	H5-1B12	(36)
mAb 12D1	^422^RIENLNKKMEDGFLDVWTYNAELLVLMENERTLDFHDSNVKNLYDKVRLQLRDNA^476^	^427^KKMEDGFLDV WTYNAELLV^446^ ^449^ENERTLDFHDSNVKN LYDK^467^	H5-LAH-1H5-LAH-2	(21)

a Previously reported monoclonal antibodies against each highly conserved B cell epitope in the HA2 polypeptide.

b We synthesized previously reported B cell epitope sequences from the HA2 polypeptide to prepare a complex containing each epitope and Lipoplex(O) in this study.

c Abbreviations of each B cell epitope used in this study.

**Figure 2:**
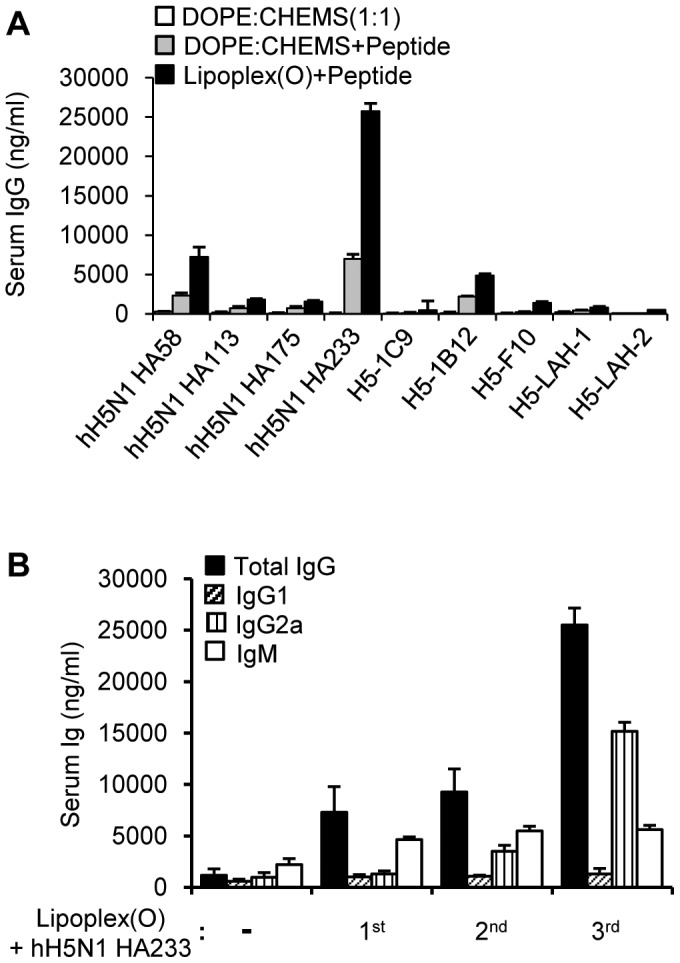
B cell epitope selection from the HA protein of the H5N1 A/Vietnam/1203/2004 strain. (**A**) BALB/c mice (N = 3/group) were injected i.p. with a DOPE:CHEMS (1∶1 ratio) complex, each peptide encapsulated in the DOPE:CHEMS complex (DOPE:CHEMS + peptide), or each peptide and Lipoplex(O) (Lipoplex(O)+peptide) on three occasions. The antisera were collected, and then amounts of each peptide-specific total IgG were assayed by ELISA. (**B**) Kinetics of IgG production in response to immunization with the complex of hH5N1 HA233 peptide and Lipoplex(O). Three BALB/c mice were injected i.p. with hH5N1 HA233 peptide and Lipoplex(O) on three occasions. The sera were collected one day before each injection and 10 days after the final injection, and amounts of the hH5N1 HA233 peptide-specific total IgG, IgG1, IgG2a and IgM were assayed by ELISA.

When we immunized BALB/c mice with the complex of each epitope from HA1 and Lipoplex(O), the BALB/c mice induced hH5N1 HA58 and hH5N1 HA233 peptide-specific IgG production more markedly than other control mice (Figure 2A). The immunization with a complex of epitope and Lipoplex(O) produced a much higher level of IgG2a than IgG1 in the mice, suggesting the induction of a Th1-dominated humoral response in animal experiments (Figure 2B). We also found that a larger amount of peptide-specific IgG (IgG2a) was produced in the secondary and tertiary responses (Figure 2B). These results suggest that the complex of B cell epitope and Lipoplex(O) induced epitope-specific IgG production and that Lipoplex(O) is important for producing an epitope-specific Th1-dominant humoral immune response.

To further evaluate the adjuvant activity of Lipoplex(O), we immunized the mice with the complex of previously reported B cell epitopes from HA2 and Lipoplex(O). However, no production of the peptide-specific IgG was observed (Figure 2A). Considering that other investigators confirmed that these epitopes induced production of HA-specific IgG [22], [35]–[37], we carefully suggest that IgG production induced by a complex of B cell epitope and Lipoplex(O) is dependent on the a novel mechanism different from that by conventional immunization method with a different preference for antigenic epitopes.

### Effect of Liposome Composition, CG Dinucleotide and Backbone Modification on IgG Production Induced by a Complex of Epitope and CpG-DNA Co-encapsulated in Liposome

**Figure 3:**
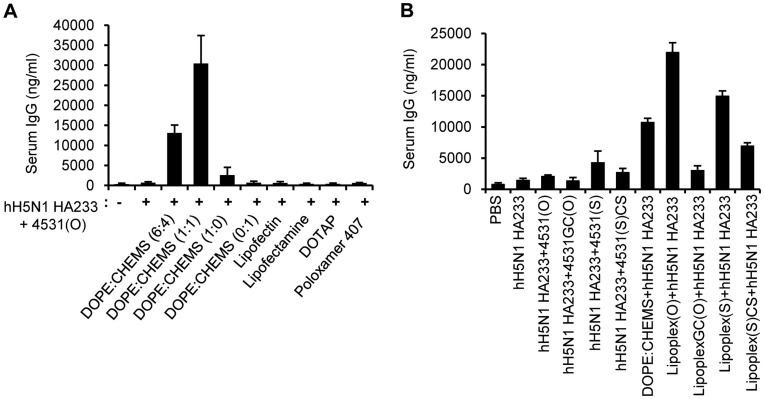
Effect of liposome composition, CG dinucleotide and backbone modification on antibody production. (**A**) Effect of the liposome composition. Three BALB/c mice were injected i.p. with a complex of hH5N1 HA233 and MB-ODN 4531(O) co-encapsulated in indicated liposomes on three occasions. The antisera were collected, and then amounts of hH5N1 HA233 epitope-specific total IgG were measured by ELISA. (**B**) Effect of CG dinucleotide and phosphorothioate backbone modification. Three BALB/c mice were injected i.p. with hH5N1 HA233 and DOPE:CHEMS (1∶1 ratio)-co-encapsulated MB-ODN 4531(O) (Lipoplex(O)+hH5N1 HA233), MB-ODN 4531GC(O) (LipoplexGC(O)+hH5N1 HA233), MB-ODN 4531(S) (Lipoplex(S)+hH5N1 HA233), and complementary MB-ODN 4531(S) (Lipoplex 4531(S)CS+hH5N1 HA233) on three occasions. The antisera were collected, and amounts of hH5N1 HA233-specific total IgG were measured by ELISA.

We compared the effect of each liposome on the IgG production in BALB/c mice after an injection of hH5N1 HA233 peptide and MB-ODN 4531(O) co-encapsulated in different liposomes. The complex of hH5N1 HA233 and MB-ODN 4531(O) co-encapsulated in DOPE:CHEMS induced more robust production of the epitope-specific IgG than the complex of hH5N1 HA233 peptide and MB-ODN 4531(O) co-encapsulated in other liposomes (Figure 3A). When we modulated the composition of DOPE:CHEMS, the highest production of serum IgG was seen with DOPE:CHEMS (1∶1 ratio) which was adopted for Lipoplex(O). To estimate the effect of the CG sequence and phosphorothioate backbone modification of MB-ODN 4531 on hH5N1 HA233 specific-IgG production in mice, we injected BALB/c mice i.p. with MB-ODN 4531(O), MB-ODN 4531GC(O), MB-ODN 4531(S) or MB-ODN 4531(S)CS (Table 3) and a complex of hH5N1 HA233 peptide co-encapsulated in DOPE:CHEMS on three occasions at 10 day intervals. MB-ODN 4531GC(O) has a reversal sequence of three CG dinucleotides, and MB-ODN 4531(S)CS sequence is a complementary sequence of MB-ODN 4531(S). As indicated in Figure 3B, the hH5N1 HA233 peptide-specific IgG production was highly induced in the mice immunized with hH5N1 HA233 peptide plus Lipoplex(O) or Lipoplex(S) which suggests that peptide-specific IgG production is independent from backbone modification. However, the IgG production was not observed in the mice immunized with hH5N1 HA233 peptide plus LipoplexGC(O) implying its dependence on CG-sequence. Basal IgG production was observed in the mice injected with the MB-ODN 4531(S)CS sequence.

**Table 3:** Synthetic ODN derivatives.

ODNs	Sequences	Modification
MB-ODN 4531(O)	AGCAGCGTTCGTGTCGGCCT	None
MB-ODN 4531GC(O)	AGCAG**GC**TTCGTGTCGGCCT	None
MB-ODN 4531(S)	AGCAGCGTTCGTGTCGGCCT	S
MB-ODN 4531(S)CS	AGGCCGACAAGAACGCTGCT	S

The MB-ODN 4531 sequences used in this study were either a natural phosphodiester bond (O) or a phosphorothoiate-modified backbone (S). The phosphorothioate version of MB-ODN 4531(O) is MB-ODN 4531(S). The change of CG dinucleotide to GC is indicated in bold letter. MB-ODN 4531(S)CS is the complementary sequences of the MB-ODN 4531(S).

### Efficiency of the Epitope-specific IgG Production by a Complex of Epitope and Lipoplex(O)

**Figure 4:**
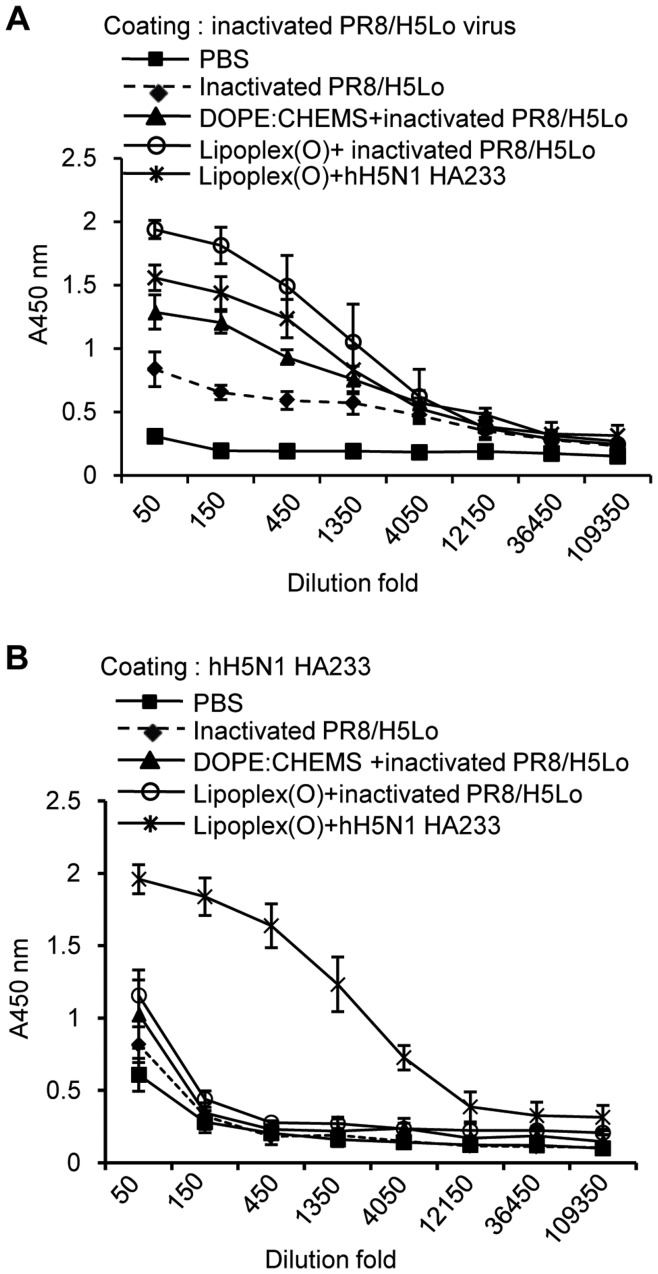
Lack of production of hH5N1 HA233-specific antibodies by an inactivated rH5N1 virus. BALB/c mice were injected i.p. with UV-inactivated rH5N1 virus, a complex of the UV-inactivated rH5N1 virus encapsulated in DOPE:CHEMS, or a complex of the UV-inactivated rH5N1 virus and Lipoplex(O) on three occasions. The sera were collected, and the titers of rH5N1 virus-specific total IgG (**A**) and hH5N1 HA233-specific total IgG (**B**) were measured by ELISA.

We compared the efficiency of the epitope-specific IgG production between whole virus immunization and a complex of epitope and Lipoplex(O) immunization in BALB/c mice. We observed a higher increase in the amount of total IgG titer against an inactivated virus in mice immunized with a complex of the inactivated rH5N1 virus and Lipoplex(O) than in mice immunized with hH5N1 HA233 peptide plus Lipoplex(O), free inactivated rH5N1 virus, or a complex of an inactivated rH5N1 virus encapsulated with DOPE:CHEMS (Figure 4A). However, no production of IgG against hH5N1 HA233 were found in the mice (Figure 4B), which suggests that the antibodies that recognize hH5N1 HA233 cannot be produced by immunization with an inactivated rH5N1 virus. These results also suggest that a complex of epitope and Lipolpex(O) may give a breakthrough when antibody production is not efficiently obtained by whole virus or protein antigens.

### Comparison of the B cell Epitope HA233 from the HA Proteins of Divergent H1N1 and H5N1 Strains

**Figure 5:**
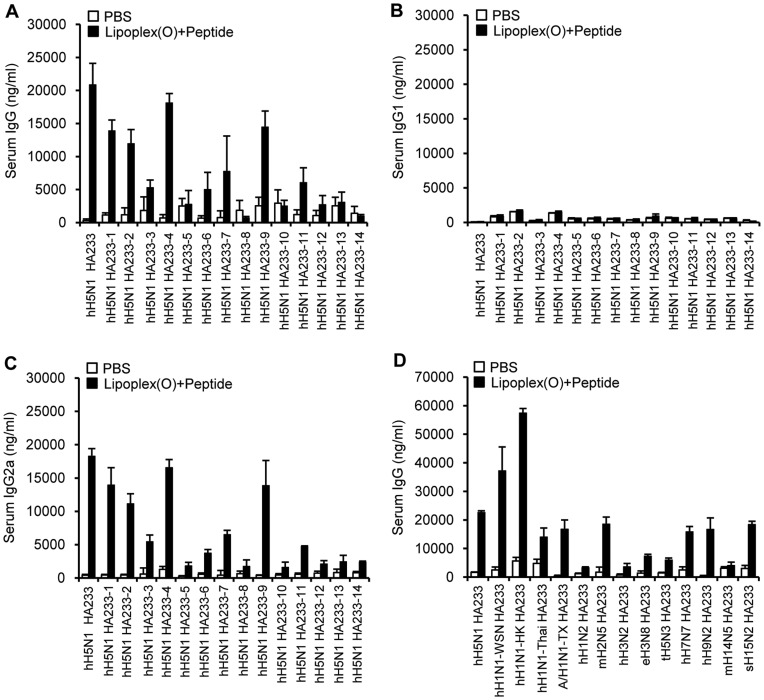
IgG production by a complex of Lipoplex(O) and conserved sequences corresponding to hH5N1 HA233 epitope. Fourteen amino acid long conserved sequences were synthesized; the sequences correspond to hH5N1 HA233 epitope of the A/Vietnam/1203/2004 strain; they were obtained from influenza A virus H5N1 strains and influenza A virus subtypes. Three BALB/c mice were immunized i.p. three times with a complex of each peptide and Lipoplex(O). The antisera were collected 10 days after the final immunization, and then amounts of each peptide-specific total IgG (**A,D**), IgG1 (**B**) and IgG2a (**C**) were measured by ELISA. The ELISA plates were coated with hH5N1-HA 233 peptide.

Of the selected B cell epitopes from the HA protein of the H5N1 A/Vietnam/1203/2004 strain, the hH5N1 HA233 epitope induces the highest epitope-specific antibody production (Figure 2). Therefore, we analyzed the hH5N1 HA233 epitope and corresponding sequences among H5N1 strains. Table 4 shows that only one or two amino acids are different between the hH5N1 HA233 epitope and each corresponding epitope of the other H5N1 strains. In contrast, we observed many different kinds of variations compared with the hH5N1 HA233 epitope in the corresponding sequences of HA proteins derived from various influenza A H1N1 virus strains (Table 5). The amount of produced IgG specific to the hH5N1 HA233 epitope or to each corresponding epitope (Table 4) was largely influenced by the amino acid sequences (Figure 5A) and the IgG2a isotype was produced at a much higher level than IgG1 (Figure 5B, 5C), suggesting a Th1-dominated humoral response. In addition, we randomly selected HA233 epitopes from from various influenza A H1N1 virus strains (Table 5) and analyzed the production of the epitope-specific IgG in mice immunized with a complex of each epitope and Lipoplex(O). These epitopes were also effective in producing each type of epitope-specific IgG depending on the amino acid sequences (Figure 5D).

**Table 4:** Sequence alignment of hH5N1 HA233 epitope from A/Vietnam/1203/2004 and corresponding sequences from other H5N1 strains.

Strain (isolation no.)^a^	Accession No.	Abbreviation	Sequences
A/Vietnam/1203/2004 (259)	AAW80717	hH5N1 HA233	IATRSKVNGQSGRM
A/Hong Kong/482/97 (21)	AAC32100	hH5N1 HA233-1	IATR**P**KVNGQSGRM
A/Hong Kong/213/03 (9)	BAE07201	hH5N1 HA233-2	IATRSKVNGQ**N**GRM
A/Indonesia/TLL007/2006 (4)	ABW74707	hH5N1 HA233-3	**M**ATRSKVNGQSGRM
A/Viet Nam/JP4207/2005 (3)	ABO10183	hH5N1 HA233-4	IATRSKVNGQSGR**I**
A/Hong Kong/483/97 (3)	AAC32099	hH5N1 HA233-5	IATR**P**KVNGQSGR**I**
A/Egypt/14724-NAMRU3/2006 (2)	ABM54179	hH5N1 HA233-6	IATRSK**I**NGQSGR**I**
A/Viet Nam/CL100/2004 (3)	ABE97630	hH5N1 HA233-7	IATRSK**I**NGQSGRM
A/Anhui/2/2005 (2)	ABD28181	hH5N1 HA233-8	IATRSKVNG**R**SGRM
A/Egypt/0636-NAMRU3/2007 (1)	ABM92273	hH5N1 HA233-9	IA**A**RSKVNGQSGRM
A/Thailand/1(KAN-1A)/2004 (1)	ABL10088	hH5N1 HA233-10	IATRS**E**VNGQSGRM
A/Egypt/2289-NAMRU3/2008 (1)	ACI06181	hH5N1 HA233-11	IATRSKVNGQ**I**GRM
A/Egypt/3300-NAMRU3/2008 (1)	ACI06185	hH5N1 HA233-12	IATRSKVNGQSGR**V**
A/Anhui/T2/2006 (2)	ABU80630	hH5N1 HA233-13	IATR**T**KVNGQSGRM
A/Indonesia/CDC1032N/2007 (1)	ABM90489	hH5N1 HA233-14	**T**ATRSKVNGQSGRM

a Numbers in parentheses denote the number of strains containing the specific sequences among the 313 different strains of human H5N1 virus isolated up to date (http://www.ncbi.nlm.nih.gov/genomes/FLU/).

**Table 5:** Alignment of the sequences corresponding to H5N1 HA233 epitope in influenza A virus subtypes.

Strains (subtypes)	Accession No.	Abbreviation	Sequences
A/Vietnam/1203/2004 (H5N1)	AAW80717	hH5N1 HA233	IATRSKVNGQSGRM
A/WSN/1933 (H1N1)	AAA43209	hH1N1-WSN HA233	IA**A**R**P**KV**KD**Q**H**GRM
A/Hong Kong/1131/1998 (H1N1)	AAK70451	hH1N1-HK HA233	IA**K**R**P**KV**RD**Q**E**GR**I**
A/Thailand/271/2005 (H1N1)	ABK57093	hH1N1-Thai HA233	IA**K**R**P**KV**R**GQ**A**GRM
A/Korea/01/2009(H1N1)	ACQ84451	hH1N1-KORHA233	IA**I**R**P**KV**RD**Q**E**GRM
A/Texas/05/2009 (H1N1)	ACP41934	A/H1N1-TX HA233	IA**I**R**P**KV**RD**Q**E**GRM
A/Michigan/2/2003 (H1N2)	ABI96104	hH1N2 HA233	I**TK**R**P**KV**RD**Q**E**GR**I**
A/Mallard/Alberta/202/96 (H2N5)	AAT65325	mH2N5 HA233	IATR**P**KVNGQ**G**GRM
A/Hong Kong/1143/99 (H3N2)	AAK62039	hH3N2 HA233	I**GS**R**PW**V**R**G**V**S**S**R**I**
A/Equine/Jilin/1/1989 (H3N8)	AAA43151	eH3N8 HA233	I**GS**R**PW**V**R**GQSGR**V**
A/Tern/South Africa/61 (H5N3)	ABI84970	tH5N3 HA233	IATR**P**KVNGQSGR**V**
A/Canada/rv504/2004 (H7N3)	ABI85000	hH7N3 HA233	**PGA**R**PQ**VNGQSGR**I**
A/England/268/1996(H7N7)	AAC40998	hH7N7 HA233	**PGA**R**PQ**VNGQSGR**I**
A/Hong Kong/1074/99 (H9N2)	CAB95857	hH9N2 HA233	I**GP**R**PL**VNG**LQ**GR**I**
A/Mallard/Astrakhan/263/1982 (H14N5)	ABI84453	mH14N5 HA233	I**GS**RP**R**V**RN**QSGR**I**
A/Shelduck/WA/1756/1983 (H15N2)	ABB90704	sH15N2 HA233	**PGA**R**P**KVNGQ**A**GR**I**

### Specificity and Cross-reactivity of IgG Produced by a Complex of Epitope and Lipoplex(O)

**Figure 6:**
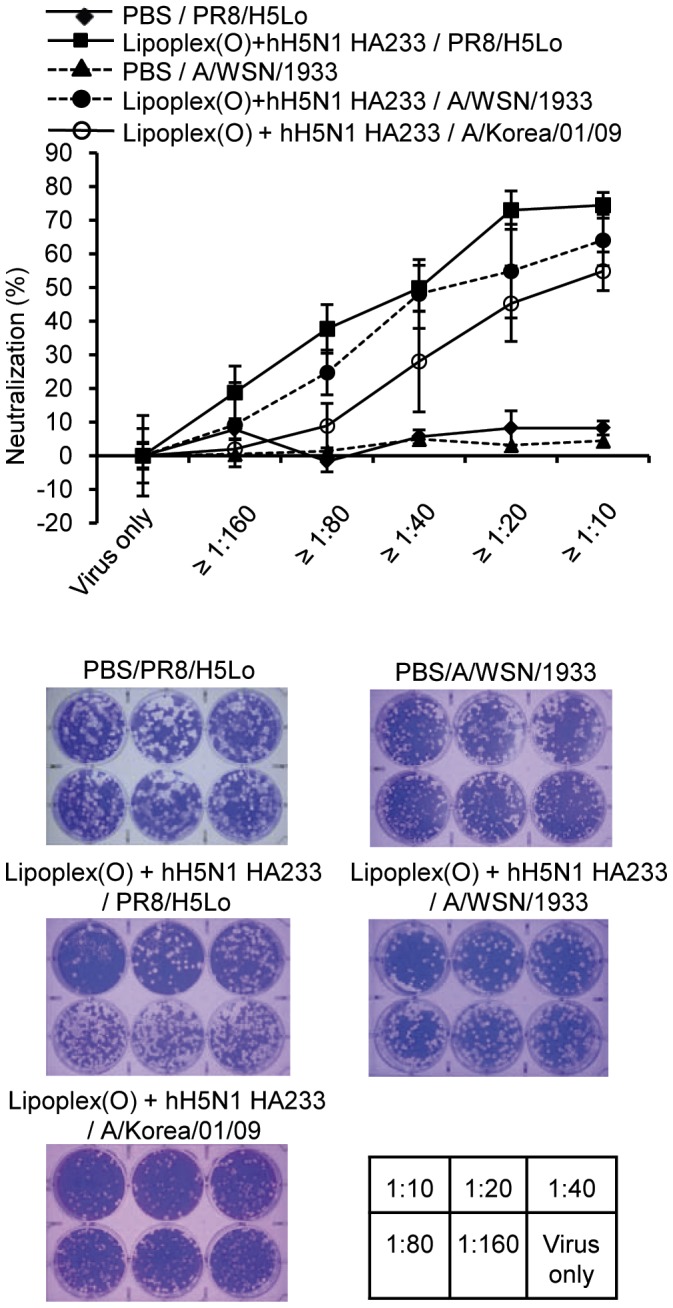
Effects of epitope-specific antibodies on influenza A viruses. Virus neutralization assays were performed with antisera collected from mice immunized with a complex of hH5N1 HA233 and Lipoplex(O). The plaque number was counted and the neutralization percentage was calculated.

To verify whether the epitope-specific antibodies produced by the complex of epitope and Lipoplex(O) could react with influenza A viruses, we performed a virus neutralization assay. The results were quantified against the recombinant H5N1 A/Vietnam/1203/2004 virus (rH5N1 virus) which was generated by reverse genetics, H1N1 virus (A/WSN/1933 virus), or swine origin H1N1 virus (A/Korea/01/09). The antisera from the mice immunized with the hH5N1 HA233 epitope efficiently neutralized the rH5N1 virus (Figure 6). The cross-reactivity of the antisera to the A/WSN/1933 virus and A/Korea/01/09 virus was detectable but lower than against the rH5N1 virus (about 70∼80%) (Figure 6).

This outcome indicates that the antisera obtained by hH5N1 HA233 immunization were cross-reactive with the corresponding epitopes of H1N1 influenza strains. It also indicates that the extent of the cross-reactivity depends on amino acid sequences of the specific epitopes.

### Prophylactic Efficacy of Vaccine Containing an Epitope and Lipoplex(O) Complex Against an Influenza A Virus Infection in Mice

**Figure 7:**
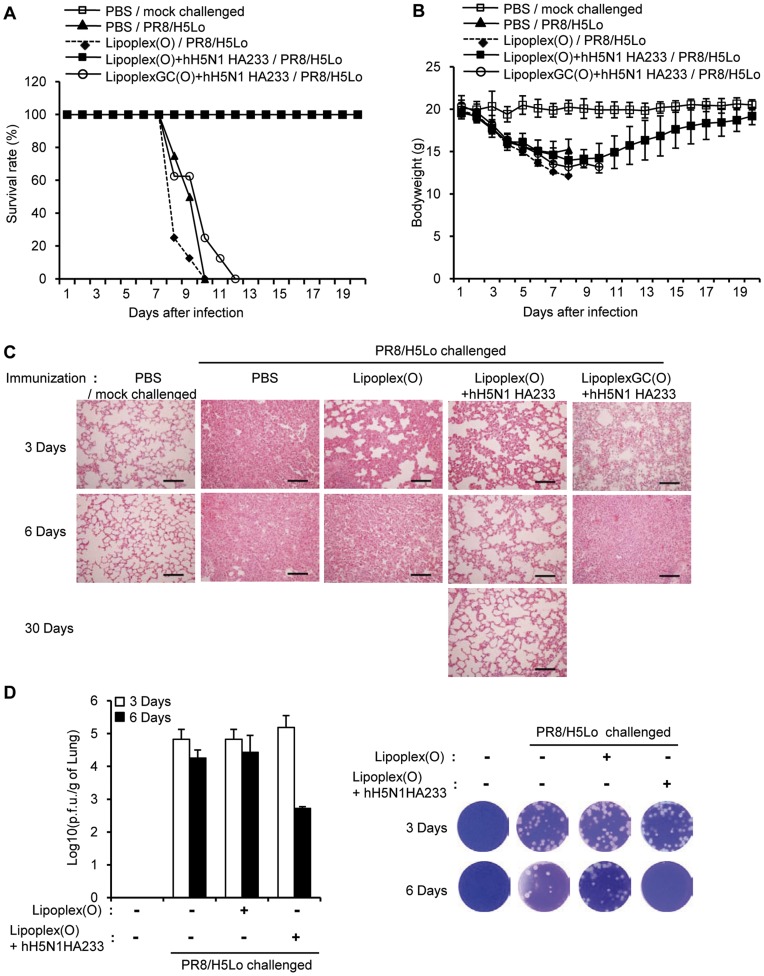
Prophylactic efficacy of a complex of hH5N1 HA233 and Lipoplex(O) against influenza A virus. BALB/c mice were immunized i.p. twice with a complex of hH5N1 HA233 encapsulated in indicated combination. The immunized mice were challenged intranasally with the rH5N1 virus (PR8/H5Lo) (**A–C**). After the virus challenge, the survival rate (**A**) and the body weight (**B**) were recorded for 20 days (N = 8/group). The lungs were collected at 3 days, 6 days or 30 days after the challenge with the rH5N1 virus (PR8/H5Lo) (**C**) (N = 3/group). Scale bars in (**C**), 100 µm. To estimate the viral clearance, the lung viral titers were measured by means of a plaque assay at 3 days or 6 days after the challenge with the rH5N1 virus (**D**). Lipoplex(O), MB-ODN 4531(O) encapsulated in DOPE:CHEMS (1∶1 ratio) complex; LipoplexGC(O), MB-ODN 4531GC(O) encapsulated in DOPE:CHEMS (1∶1 ratio) complex.

To check whether a vaccine containing a complex of hH5N1 HA233 and Lipoplex(O) has prophylactic effects, we challenged the BALB/c mice with the rH5N1 virus ten days after the vaccination. As a negative control, two groups of mice were immunized with hH5N1 HA233 and MB-ODN 4531GC(O) co-encapsulated in DOPE:CHEMS (LipoplexGC(O)+hH5N1 HA233) or with MB-ODN 4531(O) encapsulated in DOPE:CHEMS (Lipoplex(O)). The mice immunized with a vaccine containing a complex of hH5N1 HA233 and Lipoplex(O) showed a 100% survival rate, whereas all the mice of the negative control groups died (Figure 7A). All the mice immunized with a complex of hH5N1 HA233 and Lipoplex(O) had regained weight and recovered from clinical symptoms of disease by the end of the observation time (Figure 7B). We examined the lung histopathology of rH5N1 virus–infected mice 3 days and 6 days after infection and found pulmonary lesions consisting of severe necrotizing bronchitis and severe histiocytic alveolitis. The mice immunized with a complex of hH5N1 HA233 and Lipoplex(O) had significantly moderate pathological changes in the lung (Figure 7C), and the viral clearance in the lung (Figure 7D). To examine whether immunization with a complex of hH5N1 HA233 and Lipoplex(O) elicits a prophylactic effect on other influenza subtype, we challenged the BALB/c mice with the mouse-adapted H1N1 virus (maA/WSN/1933) 10 days after vaccination. The survival rate implies that a 50% survival rate was seen upon the challenge of the maA/WSN/1933 virus (Figure S1). Considering the antibody production levels in Figure 2B, significantly higher level of specific Ab was observed after third immunization. Therefore, we additionally performed the virus challenge experiments after third immunization and obtained similar results as shown in Figure S2.

Phosphorothioate-modified CpG-DNA (PS-ODN) has been studied for prophylactic application against infectious diseases in mice. Several investigations reported that mice pre-treated with PS-ODN were completely or partially protected against influenza A virus challenge depending on the periods and routes of PS-ODN pre-treatment [38], [39]. Therefore, challenge at day 10 after the immunization may be considered to be too early since innate immunity stimulated by the immunization with the adjuvant-antigen complex could readily provide partial protection against the infection. Therefore, we first injected mice with PS-ODN (CpG-ODN 1826(S)) or Lipoplex(O) and checked expression of IFN-α and IL-12 using ELISA assays. PS-ODN and Lipoplex(O) induced secretion of IFN-α and IL-12 transiently but the cytokine levels declined to basal level after 10 days (Figure S3). Therefore, the effects of innate immunity stimulated by CpG-DNA or liposome complex seem to be minimal at 10 days after pre-treatment.

**Figure 8:**
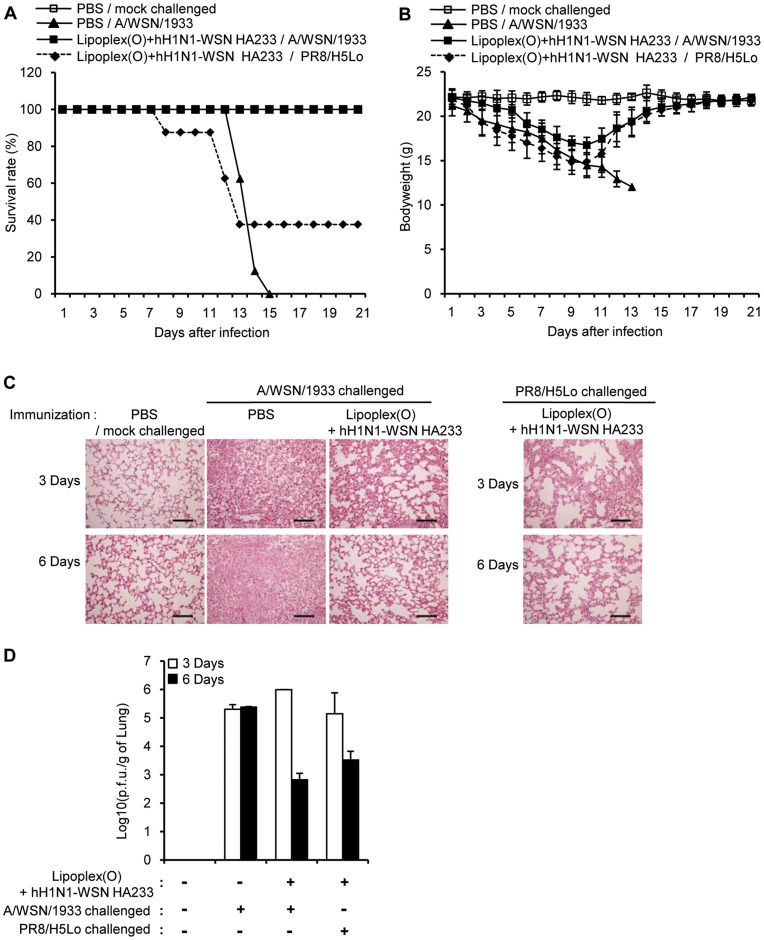
Prophylactic efficacy of a complex of hH1N1-WSN HA233 and Lipoplex(O) against influenza A virus. BALB/c mice were immunized i.p. two times with a complex of hH1N1-WSN HA233 and Lipoplex(O) (Lipoplex(O)+hH1N1-WSN HA233). The immunized mice were challenged intranasally with the rH5N1 virus (PR8/H5Lo) or the maA/WSN/1933 virus. After the virus challenge, the survival rate (**A**) and the body weight (**B**) were recorded for 22 days (N = 8/group). Lungs were collected at 3 days or 6 days after the challenge with the rH5N1 virus or the maA/WSN/1933 virus (N = 3/group) (**C**). Scale bars in (**C**), 100 µm. The lung viral titers were measured by means of a plaque assay to estimate the viral clearance at 3 days or 6 days after the challenge with the maA/WSN/1933 virus or rH5N1 virus (**D**).

**Figure 9:**
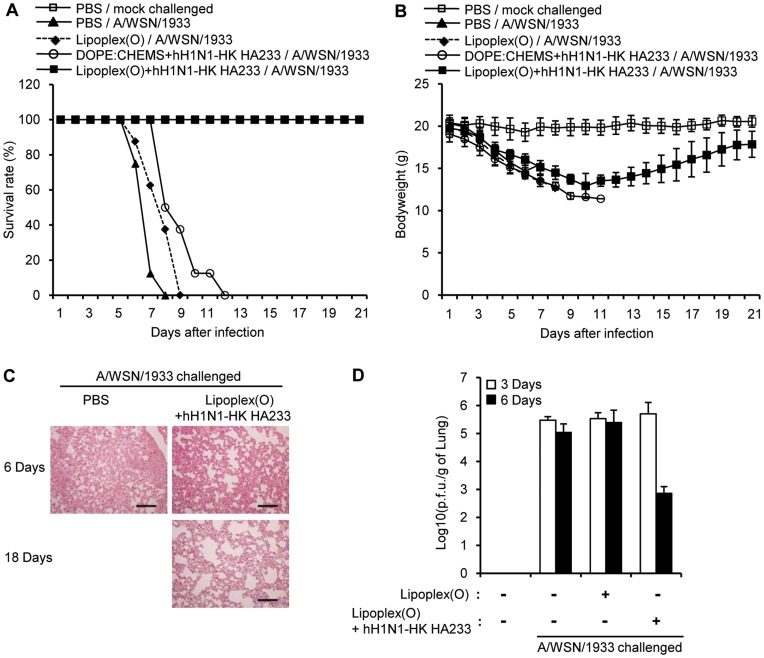
Prophylactic efficacy of a complex of hH1N1-HK HA233 and Lipoplex(O) against maA/WSN/1933 virus. BALB/c mice were immunized i.p. two times with a complex of MB-ODN 4531(O) encapsulated in DOPE:CHEMS (Lipoplex(O)), a complex of hH1N1-HK HA233 encapsulated in DOPE:CHEMS (DOPE:CHEMS+hH1N1-HK HA233), or a complex of hH1N1-HK HA233 and Lipoplex(O) (Lipoplex(O)+hH1N1-HK HA233). The immunized mice were challenged intranasally with the maA/WSN/1933 virus. After the virus challenge, the survival rate (**A**) and the body weight (**B**) were recorded for 21 days (N = 8/group). Lungs were collected at 3 days or 6 days after the challenge with the maA/WSN/33 virus (N = 3/group) (C). Scale bars in (**C**), 100 µm. The lung viral titers were measured by means of a plaque assay to estimate the viral clearance at 3 days or 6 days after the challenge with the maA/WSN/1933 virus (**D**).

We next investigated the prophylactic efficacy of a vaccine containing other B cell epitopes (hH1N1-WSN HA233 and hH1N1-HK HA233). The hH1N1-WSN HA233 vaccine was prophylactically effective against the maA/WSN/1933 virus challenge (a 100% survival rate), but less effective against the rH5N1 virus challenge with a survival rate of 40% (Figure 8). The hH1N1-HK HA233 vaccine also showed prophylactic efficacy against the maA/WSN/1933 virus challenge (a 100% survival rate) (Figure 9).

## Discussion

Chemically inactivated viral vaccines are widely used in clinics for protecting infectious and malignant diseases. However, viral vaccines have disadvantages, such as the risk of virus reactivation, the cost of maintaining vaccine stability, the induction of autoimmune diseases, and deficient protection in some vaccines [20], [40]–[42]. To overcome these disadvantages, researchers have developed synthetic epitope-based peptide vaccines over the last 30 years. To improve the efficacy of peptide vaccines for practical application, we used Lipoplex(O) as an efficient adjuvant for peptide without any additional carrier; we then successfully screened the potent peptide-based epitopes and developed efficient prophylactic vaccines against the influenza A virus by using a complex of viral epitopes and Lipoplex(O).

Phosphorothioate-modified CpG-DNA (PS-ODN), which are resistant to nuclease activity and can be efficiently delivered into cells [43], [44], have been considered in therapeutic applications [45]. The immunostimulatory activities of PS-ODN gained attention as a potentially useful form of therapeutics for immunoadjuvants [28]. However, PS-ODN induces CG sequence–independent and backbone-related side effects [46], [47]. In previous studies, we suggested that PO-ODN may be more optimal than PS-ODN for enhancing innate immune responses [29], [30]. However, the immunostimulatory effects of PO-ODN are updated only in mouse cells and not in human cells [48]. Here, we have shown that encapsulation with the DOPE:CHEMS complex enhanced the adjuvant effect of PO-ODN (MB-ODN 4531(O)) on the production of the B cell epitope–specific antibody and the efficacy of the prophylactic vaccine against influenza A virus. We successfully identified the B cell epitope H5N1 HA233 out of 4 putative epitopes from the HA protein of the H5N1 A/Vietnam/1203/2004 strain by using a complex of each predicted epitope and Lipoplex(O) (Figure 2). A complex of the epitope and Lipoplex(O) induced significantly high levels of an epitope-specific Th1-dominated humoral response in animal experiments in a DOPE:CHEMS composition (1∶1 ratio)-dependent, CpG sequence-dependent, and backbone modification-independent manner (Figure 3, Figure 5).

Divergent strains of avian and human H5N1 influenza A virus have been reported since 1997. It is important, therefore, for the prompt application, to develop the epitope-based peptide vaccine against pandemic influenza. BALB/c mice immunized with a complex of hH5N1 HA233 epitope and Lipoplex(O) produced the epitope-specific IgG (Figure 2, Figure 5), and the produced antibodies were reactive with the rH5N1 virus (Figure 6). We found that the hH5N1 HA233 epitope is relatively conserved in most influenza H5N1 strains except for a few other H5N1 strains with only one or two amino acid difference (Table 4). However, there is an apparent paradox between the antibody inducing capability of the peptides (Figure 5) and some of the peptide sequences (Table 4) which cannot be explained easily. For example, immunization with the peptide hH5N1 HA233-4 results in a clear response against HA233, whereas immunization with peptide hH5N1 HA233-5 leads to poor antibody-induction. Based on the comparison of these two peptides with the hH5N1 HA233 sequence, one would conclude that the serine to proline change at position 5 is responsible for this difference. This is also likely since a proline residue usually distorts a peptide conformation significantly. However, HA233-1 which has a Proline at position 5, appears to work very well. Therefore, there may be some complex novel mechanism in recognition of the peptide sequences as a good antigen when we use our novel peptide vaccine technology.

Based on the ability of virus neutralization, sera from mice immunized with hH5N1 HA233 were partially cross-reactive to the H1N1 virus (A/WSN/1933 virus), and swine origin H1N1 virus (A/Korea/01/09) (Figure 6). The vaccination with a complex of hH5N1 HA233 and Lipoplex(O) illustrated a prophylactic effect against infection of the rH5N1 virus; the effect is confirmed by the survival rate, the regaining of weight, and the moderate lung histopathology (Figure 7). The prophylactic effect observed after second immunization was similar to that observed after third immunization even though significantly higher level of specific Ab was observed after third immunization (Figure 7 and Figure S2). These results leave the role for specific Abs in protection to be further determined. On the other hand, the hH5N1 HA233 vaccine was less effective against the H1N1 virus (A/WSN/1933 virus) challenge with a survival rate of 50% (Figure S1). Considering the cross-reactivity of the antisera to the A/WSN/1933 virus was about 70∼80% of the reactivity to the rH5N1 virus (Figure 6), the survival rate is lower than the expected. The discrepancy between *in vitro* neutralizing activity and *in vivo* protection effect suggests that other factor(s) such as innate immunity may be involved in the protection in addition to the specific Abs. Taken together, further studies are needed on the prophylactic effect of the complex of hH5N1 HA233 and Lipoplex(O) on the challenge of multiple influenza A subtypes, H1N1 strains, and various highly pathogenic avian influenza A viruses of H5N1 strains. Such studies will allow fine tuning of a potential vaccine, which in turn should lead to improvement of the vaccine program against pandemic influenza.

Furthermore, we suggest that the antibodies that recognize hH5N1 HA233 cannot be produced by immunization with an inactivated rH5N1 virus (Figure 4). This result implies that potent epitopes, which cannot be easily found using proteins or viruses as an antigen, can be screened and that epitope-specific antibodies can be produced when we use a complex of peptide epitopes and Lipoplex(O). This result also suggests a possibility that hH5N1 HA233 epitope is not under selective pressure in immunized individuals. Thus, it would be very interesting to find out if a selective pressure on the epitopes produces escape mutants; there might be antigenic variants during repeated selection process in susceptible cells *in vitro* or in immunized individuals *in vivo* when treated with the epitope-specific monoclonal antibodies. We warrant clarifying this issue in the future works. To understand the mechanism involved in the potent adjuvant activity of Lipoplex(O), we also need to clarify other important issues. Human beings have many different genetic backgrounds of HLA types. Therefore, it has to be determined whether IgG production by a complex of B cell epitope and Lipoplex(O) depends on the MHC type and Th1 differentiation and if a novel mechanism is involved.

There are several reports finding B cell epitopes of the HA2 polypeptide and producing monoclonal antibodies through phage-display library selection, HA protein immunization or long size synthetic polypeptide immunization (Figure 2, Table 2) [22],[35]–[37]. Here, we have shown that the epitopes previously used by other groups failed to induce the epitope-specific antibody production by immunization with the complex of each epitope and Lipoplex(O) (Figure 2). These experimental results again carefully suggest that antibody production with the complex of epitope and Lipoplex(O) without carriers is regulated by unknown key modulating mechanism in immune systems. The antisera against the hH5N1 HA233 epitope which includes the 220 loop of HA1 region (Figure 1B) had no HI titers (data not shown) suggesting that antisera against HA233 epitope do not block the interaction between HA and its receptor. Previously, similar results were reported that monoclonal HA1-specific antibodies had no HI activity but inhibited virus fusion activity [49]–[51].

In conclusion, we selected the specific B cell epitope hH5N1 HA233, and the vaccine complex of hH5N1 HA233 epitope and Lipoplex(O) stimulated potent protective responses in mice against infection from the rH5N1 virus. To determine the feasibility of developing a vaccine that affords efficient protection against multiple subtypes, we need to ensure that further studies focus on a complex that combines several epitopes together (for example epitopes derived from H1N1 subtypes) with Lipoplex(O).

## Materials and Methods

### Oligodeoxynucleotides and Reagents

ODNs were synthesized from Samchully Pharm (Seoul, Korea) and GenoTech (Daejeon, Korea). The potent immunostimulatory PO-ODN, MB-ODN 4531 [29], consisted of 20 bases that contained three CpG motifs (underlined): AGCAGCGTTCGTGTCGGCCT. The derivatives and backbone modification of the MB-ODN 4531 sequences and CpG-ODN 1826(S) are described in Table 3. The endotoxin content of the ODNs was less than 1 ng/mg of ODN as measured by a *Limulus amebocyte* assay (Whittaker Bioproducts, Walkersville, MD, USA).

### Selection and Synthesis of Peptides

Predicted B cell epitopes were selected on the basis of their hydrophilicity values from Parker *et al* [52], surface accessibility values from Emini *et al* [53], β-turn region values from Chou and Fasman [54], and antigenicity index from Kolaskar and Tongaonkar [55] (http://tools.immuneepitpoe.org/main/index.html) from the HA protein of the H5N1 A/Viet Nam/1203/2004 strain (NCBI database, AAW80717) (Table 1). The parameters were averaged over six amino acid residues and the regions above the threshold value 1.0 were chosen for each prediction factor. We synthesized 14 amino acid long peptides, which correspond to the potent epitope (hH5N1 HA233) of the H5N1 A/Vietnam/1203/2004 strain; they were derived from the HA proteins of several influenza A strains and subtypes (Table 4, Table 5). Peptides were synthesized by the Fmoc solid-phase method using an automated peptide synthesizer (Peptron III-R24, Peptron, Daejeon, Korea). After deprotection of the synthesized peptides from the resin, the peptides were purified and analyzed by reverse-phase HPLC (Waters 2690 Separations Module, Waters, Milford, USA) using Vydac C8 analytical RP column to a purity of greater than 90%. The peptides were identified by means of a mass spectrometer (HP 1100 Series LC/MSD, Hewlett-Packard, Roseville, USA).

### Preparation of a Complex of Peptide and CpG-DNA Coencapsulated in DOPE:CHEMS

Liposome complexes consisting of peptide and CpG-DNA co-encapsulated in DOPE:CHEMS were prepared as reported previously [56] with minor modifications [32], [33]. In brief, the DOPE and CHEMS were mixed at a molar ratio of 1∶1 in ethanol. To make a solvent-free lipid film, we evaporated the mixture with nitrogen gas and resuspended it in an equal volume of water soluble peptide and a CpG-DNA mixture with vigorous stirring at room temperature for 30 min. After adjusting the pH to 7.0, we slightly sonicated the liposome solution for 30 s with a sonicator and filtered the solution with a 0.22 µm filter; the solution was then freeze-thawed three times in liquid nitrogen.

### Mice and Immunization

Mice were maintained under a specific-pathogen-free (SPF) condition. Four-week-old male BALB/c (H-2^b^) mice were purchased from Central Lab. Animal Inc. (Seoul, Korea). All animal procedures performed in this study are in accordance with the recommendations in the Guide for the Care and Use of Laboratory Animals of the National Veterinary Research & Quarantine Service of Korea. The protocol was approved by the Institutional Animal Care and Use Committee of Hallym University (Permit Number: Hallym 2009–48). The mice were challenged with virus or sacrificed under Zoletil 50+Rompun anesthesia, and all efforts were made to minimize suffering.

The mice were injected i.p. with 50 µg of each peptide supplemented with either 50 µg of CpG-DNA or 50 µg of a complex of CpG-DNA co-encapsulated in DOPE:CHEMS on three occasions at 10 day intervals.

### Antigen-specific Ig ELISA

The mice were sacrificed 10 days after the final injection. Sera were collected from the blood samples obtained by a heart punch method or orbital bleeding and then stored at -70°C. To measure the amounts of total IgG, IgG1 and IgG2a, we coated the 96-well immunoplates (Nalgen Nunc International, Rochester, NY, USA) with 5 µg/ml of peptide and then blocked them with 0.05% of Tween-20 in PBS (PBST) containing 1% BSA. The sera were diluted to 1∶450 with PBST and added to the wells of each plate. The plates were incubated for 2 h at room temperature and washed with PBST. Then, rat anti-mouse biotinylated secondary antibodies (total IgG, IgG1, IgG2a) (BD Biosciences) were added to the wells and incubated for 1 h, followed by addition of streptavidin conjugated with horseradish peroxidase for 30 min. To establish the standard curve, purified mouse monoclonal immunoglobulin isotype (IgG1, IgG2a, IgM) standards (BD Pharmingen) were serially diluted in PBST and added to each well. After washing with PBST, biotin-labeled anti-mouse isotype (IgG1, IgG2a, IgM) specific antibody (BD Pharmingen) was added for 1 h. The plates were washed with PBST and incubated with horseradish peroxidase conjugated-streptavidin (Sigma, St. Louis, MO) for 30 min at room-temperature. A colorimetric assay was developed with a TMB substrate solution (Kirkegaard and Perry Laboratories, Gaithersburg, MD, USA), and we used a Spectra Max 250 microplate reader (Molecular Devices, Sunnyvale, CA, USA) to measure the absorbance at 450 nm.

### Cells and Viruses

Madin-Darby Canine Kideny (MDCK) cells were obtained from American Type Culture Collection. The MDCK cells were maintained in Dulbecco’s minimum essential medium supplemented with 10% FBS, 100 µg/ml streptomycin, and 100 U/ml penicillin. Stocks of a recombinant virus, the A/WSN/1933 virus, and the A/Korea/01/09 virus were prepared by inoculation into SPF embryonated chicken eggs or by infection of MDCK cells followed by incubation at 37°C for 72 h. All the experiments involving viruses were performed under a level 2 biosafety condition.

### Recombinant Virus

Segments of the A/Vietnam/1203/2004 (H5N1) and A/Puerto Rico/8/34 (PR8) (H1N1) influenza viruses were cloned into plasmids for virus rescue by reverse genetics as described previously [57]. We used the reassortant virus PR8/H5Lo, which bears the HA gene segment of avian-lineage A/Vietnam/1203/2004 and the remaining seven segments from PR8.

### Virus Titration

To determine the titers of virus, we performed plaque assays on MDCK cells as described previously [58]. Tenfold serially diluted suspension of virus stocks or lung tissue homogenates were added onto a confluent monolayer of MDCK cells in six-well plates and incubated at room temperature for 1 h for adsorption with shaking every 10 min. The suspension was removed and the cells were covered with MEM containing 2% oxoid agar, 5% NaHCO_3_, 1% DEAE Dextran, and (L-tosylamido-2-phenyl) ethyl chloromethyl ketone (TPCK, 1 µg/ml)-treated trypsin. After incubation at 37°C for 3 days, the dishes were stained with 1 ml of crystal violet for 15 min so that we could visualize the plaques. The numbers of plaques were counted to determine the titers.

### Determination of the Lethal Dose of the Influenza A Viruses

To determine the LD_50_ for each virus strain, we anesthetized BALB/c mice (N = 6/group) and inoculated them intranasally with serial dilutions of the virus. The survival rates and body weights of the infected mice were monitored every day. The LD_50_ was defined as the dilution of the virus that produced lethality in 50% of the mice, and LD_50_ titers were calculated by the method of Reed and Muench [59].

### Virus Neutralization Assay

To confirm antibodies targeting the challenging virus, we performed a virus neutralization assay. The antisera were treated with receptor-destroying enzymes (RDE; bacterial neuraminidase of *Vibrio cholerae*; Denka Seiken, Japan) overnight and then incubated at 56°C for 30 min to inactivate remaining RDE before use. Approximately 100 PFU/ml of influenza viruses (the rH5N1 virus, A/WSN/1933 and A/Korea/01/09 virus) were incubated with an equal volume of heat-inactivated twofold serially diluted serum samples at 37°C for 1 h. After incubation, the mixtures were added to a confluent monolayer of MDCK cells in a minimum essential medium supplemented with 10% FBS and TPCK-treated trypsin. The cells were cultured for 72 h before the determination of the cytopathic effect. The neutralization percentage was calculated by means of the following equation: Neutralization (%, percent inhibition)  =  [(plaque no. of the virus only treated – plaque no. of serially diluted serum mixed virus)/plaque no. of the virus only treated]×100.

### Hemagglutination Inhibition Assay

A hemagglutination inhibition assay was performed as described previously [60]. Briefly, viruses (the rH5N1 virus, A/WSN/1933 and A/Korea/01/09 virus) were diluted to 4 HA units and incubated with an equal volume of serial twofold dilutions of receptor-destroying enzyme-treated serum samples for 1 h at room temperature. An equal volume of 0.5% chicken red blood cells was added to the wells and incubated for 30 min to measure the HI titers.

### Inactivation of Viral Infectivity by UV Exposure

For UV-induced inactivation of the rH5N1 virus (PR8/H5Lo), the virus was irradiated at a distance of 5 cm with 1500 µM/s/cm^2^ UV for 5 min. To confirm the virus inactivation, we incubated MDCK cells with the inactivated virus (5 µg total viral proteins) for 3 days at 37°C in the presence of 1 µg/ml TPCK-trypsin. Plaque assays of the UV-inactivated virus on the MDCK cells demonstrated a complete loss of infectivity.

### Virus Challenge Experiments

Four-week-old BALB/c mice (N = 8) were injected i.p. with 50 µg of a complex of peptide and Lipoplex(O) on two occasions at 10 day intervals. Ten days after the second immunization, the mice were intranasally challenged with the 10 LD_50_ maA/WSN/1933 virus or the 10 LD_50_ rH5N1 virus. After being infected, the mice were monitored daily for clinical signs and body weight. At the indicated times, the mice were sacrificed by CO_2_ and all their organs were removed. Organ homogenates were prepared, and the virus titers of each organ were determined by means of a plaque assay. For a histopathologic examination, the lungs were removed and fixed in a 4% buffered formalin solution; they were then embedded in paraffin by a conventional method and cut into 4 µm thick sections. The specimens were stained with hematoxylin and eosin.

### Accession Numbers Mentioned in Text

A/Vietnam/1203/2004 H5N1 HA nucleotide sequence – NC_ AY818135.1.

A/Vietnam/1203/2004 H5N1 HA protein – NC_ AAW80717.

A/Hong Kong/485/1997 H5N1 HA protein – NC_AAD52043.

A/WSN/1933 H1N1 HA protein – NC_AAA43209.

A/New York/604/1995 H1N1 HA protein – NC_ ABE11867.

A/Wisconsin/4754/1994 H1N1 HA protein – NC_AAB03291.

A/Texas/05/2009 A/H1N1 HA protein – NC_ ACP41934.

A/Hong Kong/1131/1998 H1N1 HA protein – NC_AAK70451.

A/Thailand/271/2005 H1N1 HA protein – NC_ ABK57093.

A/Michigan/2/2003 hH1N2 HA protein – NC_ ABI96104.

A/Mallard/Alberta/202/96 H2N5 HA protein – NC_ AAT65325.

A/Hong Kong/1143/99 H3N2 HA protein – NC_ AAK62039.

A/Equine/Jilin/1/1989 H3N8 HA protein – NC_ AAA43151.

A/Tern/South Africa/61 H5N3 HA protein – NC_ ABI84970.

A/England/268/1996 H7N7 HA protein – NC_ AAC40998.

A/Hong Kong/1074/99 H9N2 HA protein – NC_ CAB95857.

A/Mallard/Astrakhan/263/1982 H14N5 HA protein – NC_ ABI84453.

A/Shelduck/WA/1756/1983 H15N2 HA protein – NC_ ABB90704.

A/Duck/Singapore/3/1997 Avian H5 HA protein – NC_ABQ58920.

## Supporting Information

Figure S1 **Prophylactic efficacy of a complex of hH5N1 HA233 and Lipoplex(O) against influenza A virus.** BALB/c mice were immunized i.p. twice with a complex of hH5N1 HA233 encapsulated in indicated combination. The immunized mice were challenged intranasally with the maA/WSN/1933. After the virus challenge, the survival rate (**A**) and the body weight (**B**) were recorded for 20 days (N = 8/group). Lipoplex(O), MB-ODN 4531(O) encapsulated in DOPE:CHEMS (1∶1 ratio) complex; LipoplexGC(O), MB-ODN 4531GC(O) encapsulated in DOPE:CHEMS (1∶1 ratio) complex.
(TIF)

Figure S2 **Prophylactic efficacy of a complex of hH5N1 HA233 and Lipoplex(O) against influenza A virus.** BALB/c mice were immunized i.p. three times with a complex of hH5N1 HA233 encapsulated in indicated combination. The immunized mice were challenged intranasally with the rH5N1 virus (PR8/H5Lo) (**A–C**). After the virus challenge, the survival rate (**A**) and the body weight (**B**) were recorded for 20 days (N = 8/group). The lungs were collected at 3 days, 6 days or 30 days after the challenge with the rH5N1 virus (PR8/H5Lo) (**C**) (N = 3/group). Scale bars in (**C**), 100 µm. The lung viral titers were measured by means of a plaque assay to estimate the viral clearance from the lungs at 3 days or 6 days after the challenge with the rH5N1 virus (**D**). Lipoplex(O), MB-ODN 4531(O) encapsulated in DOPE:CHEMS (1∶1 ratio) complex; LipoplexGC(O), MB-ODN 4531GC(O) encapsulated in DOPE:CHEMS (1∶1 ratio) complex.
(TIF)

Figure S3 **Production of cytokines in mice treated with CpG-DNA.** BALB/c mice (n = 3/group) were injected i.p with CpG-ODN 1826(S) or Lipoplex(O), and sera from the mice were harvested at the indicated times after injection. The concentration of IL12p40 (**A**) and IFN-α (**B**) in the serum was determined by using an ELISA assay.
(TIF)
