# Supplementary figures and images for: Immunization with a Hemagglutinin-Derived Synthetic Peptide Formulated with a CpG-DNA-Liposome Complex Induced Protection against Lethal Influenza Virus Infection in Mice
Source: PLoS One. 2012 Nov 7;7(11):e48750. doi: 10.1371/journal.pone.0048750 (PMC3492448; doi:10.1371/journal.pone.0048750)

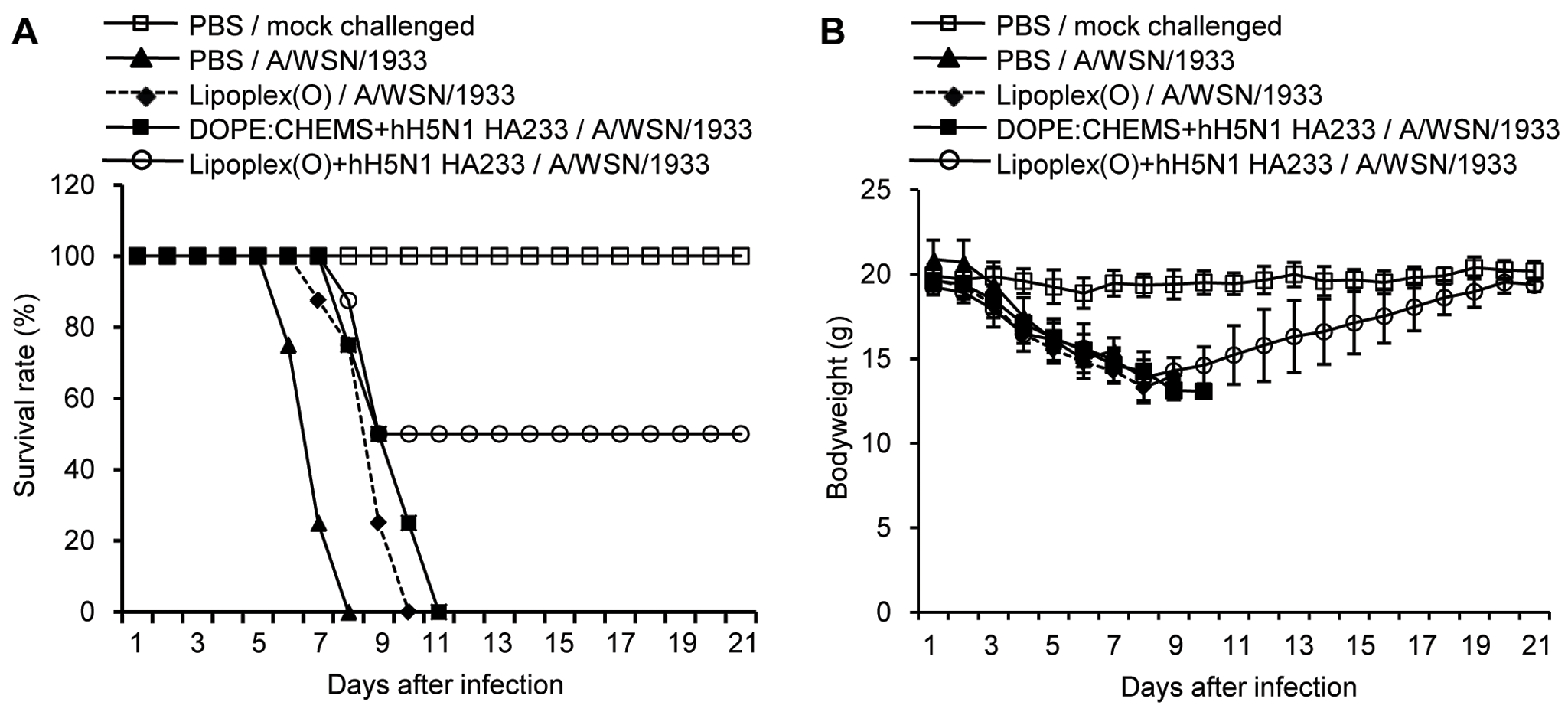

Supplement: Figure S1 — Prophylactic efficacy of a complex of hH5N1 HA233 and Lipoplex(O) against influenza A virus. BALB/c mice were immunized i.p. twice with a complex of hH5N1 HA233 encapsulated in indicated combination. The immunized mice were challenged intranasally with the maA/WSN/1933. After the virus challenge, the survival rate (A) and the body weight (B) were recorded for 20 days (N = 8/group). Lipoplex(O), MB-ODN 4531(O) encapsulated in DOPE:CHEMS (1∶1 ratio) complex; LipoplexGC(O), MB-ODN 4531GC(O) encapsulated in DOPE:CHEMS (1∶1 ratio) complex. (TIF) [file pone.0048750.s001.tif]

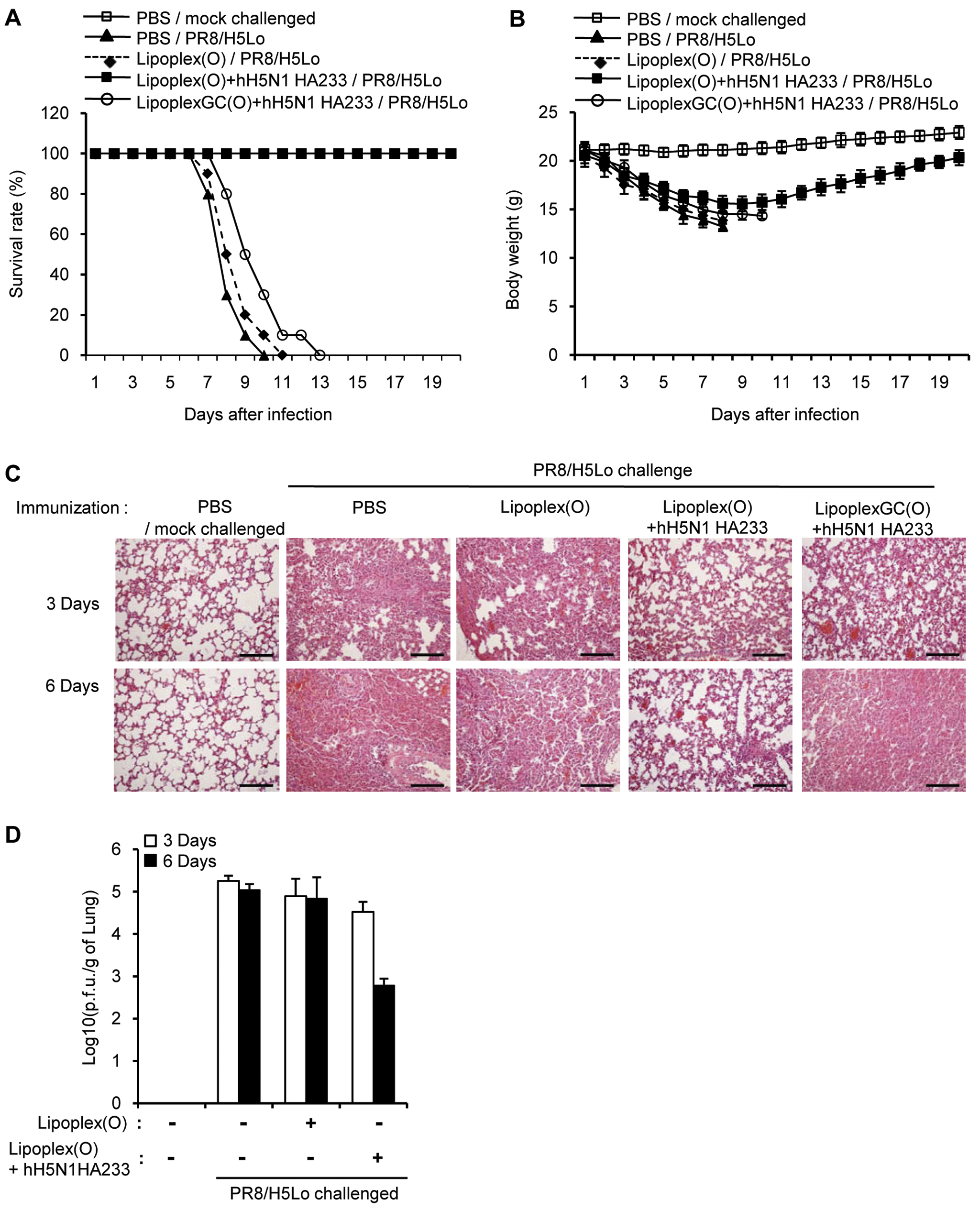

Supplement: Figure S2 — Prophylactic efficacy of a complex of hH5N1 HA233 and Lipoplex(O) against influenza A virus. BALB/c mice were immunized i.p. three times with a complex of hH5N1 HA233 encapsulated in indicated combination. The immunized mice were challenged intranasally with the rH5N1 virus (PR8/H5Lo) (A–C). After the virus challenge, the survival rate (A) and the body weight (B) were recorded for 20 days (N = 8/group). The lungs were collected at 3 days, 6 days or 30 days after the challenge with the rH5N1 virus (PR8/H5Lo) (C) (N = 3/group). Scale bars in (C), 100 µm. The lung viral titers were measured by means of a plaque assay to estimate the viral clearance from the lungs at 3 days or 6 days after the challenge with the rH5N1 virus (D). Lipoplex(O), MB-ODN 4531(O) encapsulated in DOPE:CHEMS (1∶1 ratio) complex; LipoplexGC(O), MB-ODN 4531GC(O) encapsulated in DOPE:CHEMS (1∶1 ratio) complex. (TIF) [file pone.0048750.s002.tif]

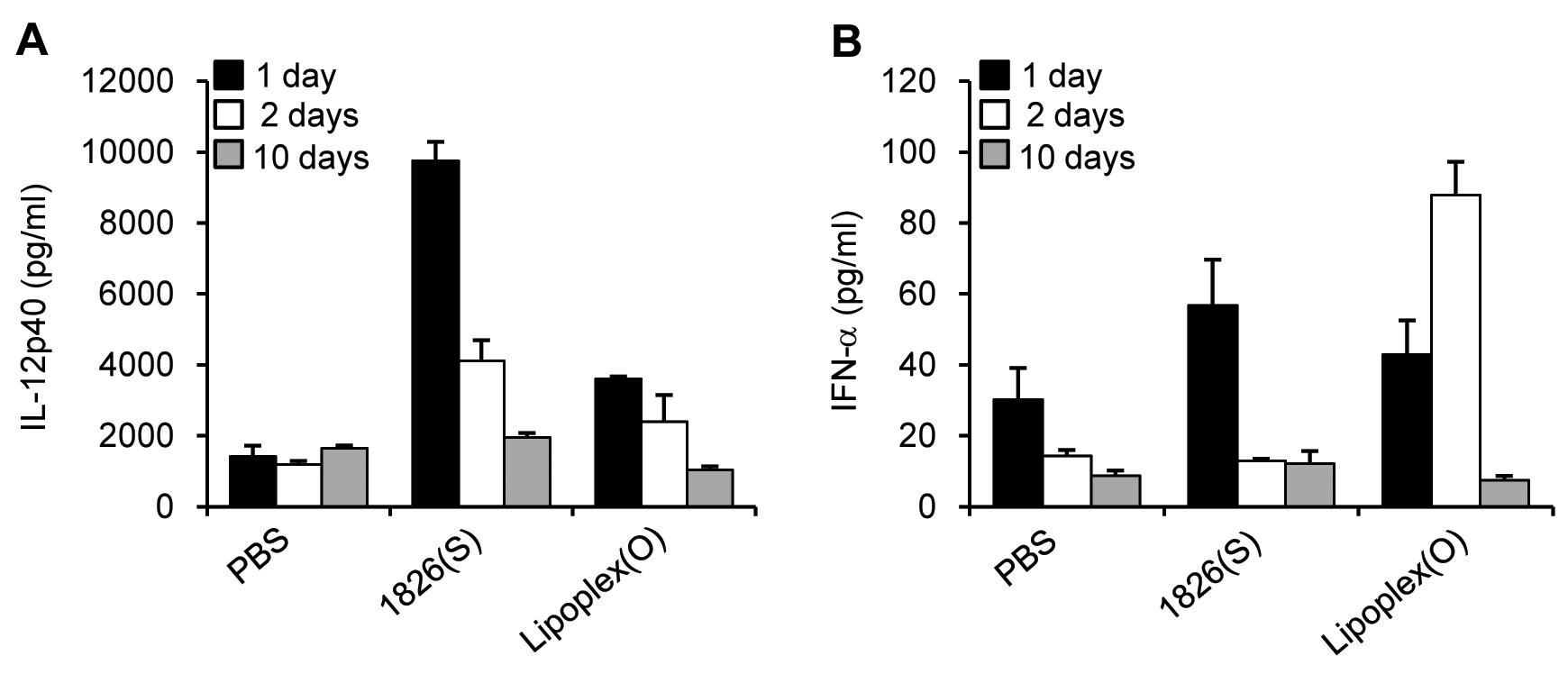

Supplement: Figure S3 — Production of cytokines in mice treated with CpG-DNA. BALB/c mice (n = 3/group) were injected i.p with CpG-ODN 1826(S) or Lipoplex(O), and sera from the mice were harvested at the indicated times after injection. The concentration of IL12p40 (A) and IFN-α (B) in the serum was determined by using an ELISA assay. (TIF) [file pone.0048750.s003.tif]
